# Preferences for health insurance in Germany and the Netherlands – a tale of two countries

**DOI:** 10.1186/s13561-014-0022-6

**Published:** 2014-10-24

**Authors:** Karolin Leukert-Becker, Peter Zweifel

**Affiliations:** 1 Polynomics AG, Baslerstrasse 44, Olten, 4600, Switzerland; 2 Emeritus, Department of Economics, University of Zurich, Kreuth 371, Bad Bleiberg, A-9531, Austria

**Keywords:** Preference measurement, Health insurance, Discrete choice experiments, Health reform, Germany, Netherlands

## Abstract

**Background:**

This contribution seeks to measure preferences for health insurance in Germany and the Netherlands, using two Discrete Choice Experiments (DCE). Since the Dutch DCE was carried out right after the 2006 health reform, which made citizens explicitly choose a health insurance contract, two research questions naturally arise. First, are the preferences with regard to contract attributes (such as Managed Care-type restrictions of physician choice), incentives (such as bonus options for no claims, deductibles, and a bonus for preventive behavior), and extra services provided by the health insurer (such as patient counseling) similar between the two countries? Second, was the requirement to explicitly choose imposed by the Dutch government in the context of the reform effective in reducing status quo bias with respect to future reforms?

**Results:**

Based on random-effects Probit estimates, these two questions can be answered as follows. First, there is resistance against Managed Care-type attributes in both populations, but Germans would have to be compensated more for giving up free physician choice. Second, their status quo bias is twice as important as among their Dutch counterparts, who apparently learned to bear the cost of information associated with future choices concerning their health insurance.

**JEL codes:**

C25, D12, I18

## Background

Governments in industrial countries have been trying to respond to the rising cost of health care by modifying health insurance (copayments, bonus options for new claims) or changing the provision of health care (Managed Care). However, it is far from clear whether citizens are ready to accept these changes. If they conceive e.g. Managed Care as constraining their choice of physician, compensation must be offered to gain their acceptance. In insurance-based systems, observed past choices provide little guidance because they are distorted by regulated contributions to health insurance, while in National Health Service-type systems, medical care has a tax price that is the same at a given income level.

In this situation, experimental evidence concerning citizens’ preferences may be of value to avoid costly mistakes by health insurers and policy makers. The present contribution purports to report on so-called market experiments of the Discrete Choice (DCE) type in two insurance-based countries, Germany and the Netherlands. This international comparison is of particular interest for at least two reasons. First, while the two populations are not too dissimilar culturally, German health policy has been characterized by new laws and regulations that have increased uncertainty on the part of patients (Böcken et al. [1]). By way of contrast, in the Netherlands a major pro-competitive reform was enacted in 2006, accompanied by a major information campaign designed to help citizens choose a health insurance contract. Second, the Dutch changes amount to something like a crossover between the two countries. The status quo in the Netherlands is gatekeeping by physicians (a variant of Managed Care), whereas consumers possibly prefer free choice of physician. In Germany, free choice of physician constitutes the status quo, but policy makers consider introducing Managed Care into social health insurance. Also, the Dutch insured were familiar with a bonus for no claims reminiscent of auto liability insurance, yet there were signs that they wanted to return to conventional health insurance with almost no copayment (Ministerie van Volksgezondheid, Welzijn en Sport [2]). In Germany, bonus options have been discussed as a reform feature. Against this backdrop, this paper seeks to answer two questions:

Q1: Are preferences of German and Dutch consumers similar or dissimilar with regard to attributes of health insurance?

Q2: Did the requirement to explicitly choose a health insurance policy imposed by the Dutch government in the context of the 2006 reform cause consumers to have less status quo bias with regard to future reforms of health insurance?

This paper is organized as follows. Section 2 is devoted to a sketch of the theory underlying DCEs. Section 3 describes the DCE and its results for Germany, while Section 4 does the same for the Netherlands. Section 5 contains a comparison of the two countries, while Section 6 offers concluding remarks.

## Methods

###  Theory underlying discrete choice experiments

Respondents participating in a Discrete Choice Experiment (DCE) are supposed to maximize (expected) utility. However, experimenters will never know all the determinants of individual utility, which therefore give rise to a degree of randomness in observed choices (Thurstone [3]). Therefore, the relevant theoretical basis is the random utility model developed by McFadden [4],[5] and Manski [6]. Let *V*_*ij*_ denote the level of utility optimally reached by individual *i* in situation *j*. Alternative *j* is associated with price *p*_*j*_, a vector of attributes *b*_*j*_ of the alternative, and income *y*_*i*_ of the individual and his or her socioeconomic characteristics *s*_*i*_. Finally, choices are also influenced by stochastic term *ε*_*ij*_ that varies between individuals and alternatives. Therefore, indirect utility is given by

(1) $$V_{\mathrm{ij}}=v\left(p_{j},b_{j},y_{i},s_{i},\varepsilon_{\mathrm{ij}}\right)\text{.}$$

The standard assumption is that this utility can be split into a deterministic and a stochastic part, with *w*(·) containing the deterministic component,

(2) $$v\left(p_{j},b_{j},y_{i},c_{i},\varepsilon_{\mathrm{ij}}\right)=w\left(p_{j},b_{j},y_{i},s_{i}\right)+\varepsilon_{\mathrm{ij}}\text{.}$$

Since for the experimenter decisions contain a stochastic element, all that can be analyzed is the probability *P*_*ij*_ of individual *i* choosing alternative *j* rather than alternative *l*. With alternative *j* by assumption yielding at least as much utility as any other alternative *l*, one has

(3) $$P_{\mathrm{ij}}=\mathrm{Pr}\left[w\left(p_{j},b_{j},y_{i},s_{i}\right)+\varepsilon_{\mathrm{ij}}\geq w\left(p_{l},b_{l},y_{i},s_{i}\right)+\varepsilon_{\mathrm{il}},\forall l\neq j\right]\text{.}$$

Rearranging yields

(4) $$P_{\mathrm{ij}}=\mathrm{Pr}\left[\varepsilon_{\mathrm{il}}-\varepsilon_{\mathrm{ij}}\leq w\left(p_{j},b_{j},y_{i},s_{i}\right)-w\left(p_{l},b_{l},y_{i},s_{i}\right),\forall l\neq j\right]\text{.}$$

The probability of choosing alternative j rather than l therefore amounts to the probability that the stochastic difference (*ε*_*il*_ – *ε*_*ij*_) is dominated by the systematic difference in utilities (*w*_*ij*_ – *w*_*j*_). This condition however can only be related to observable choices if (*ε*_*il*_ – *ε*_*ij*_) follows a distribution law. The major alternatives are the logistic and the normal. Since the normal distribution is subject to less restrictive assumptions (Train [7], ch. 7; Greene [8] ch. 19; Ben-Akiva and Lerman [9], ch. 3), this is the preferred choice (Probit model).

In the course of the experiment, every participant makes several choices. Therefore, observations are of the panel type, a fact that is reflected in the specification of the error term. Writing the difference between the two error terms as *ϑ*_*ij*_ = *ε*_*il*_ − *ε*_*ij*_, the so-called random effects specification reads (Johnson and Desvousges [10]),

(5) $$\vartheta_{\mathrm{ij}}=\upsilon_{i}+\eta_{\mathrm{ij}}\text{.}$$

In this equation, *υ*_*i*_ denotes the individual-specific component, which remains the same in the course of the experiment. By way of contrast, *η*_*ij*_ can vary between individuals *i* and scenarios *j*.

The deterministic part *w*(·) of the utility function usually is assumed to be linear and hence additively separable (Johnson and Desvousges [10]; Ryan and Gerard [11]),

(6) $$w\left(p_{j},b_{j}y_{i},s_{i}\right)=\gamma_{0}+\sum_{k=1}^{K}\gamma_{k}b_{\mathrm{jk}}+\gamma_{p}p_{j}+\gamma_{y}y_{i}+\gamma_{s}s_{i}\text{,}$$

with (*γ*_*K*_, *γ*_*p*_*γ*_*y*_*γ*_*s*_) denoting the parameters belonging to the arguments of the utility function. In particular, *γ*_*k*_ denotes the marginal utility of product attribute *k*.

Note the restrictiveness of this formulation, stating the all respondents have the same additively separable function *w*(·). Since the contribution paid for health insurance constitutes an attribute as well, is also true that the marginal loss of utility due to an increased contribution must be the same for all individuals, independently of their income. Such an assumption is deemed unrealistic because usually marginal utility of income is assumed to decrease in income. However, socioeconomic differences in marginal utility of attributes can be made part of the specification. All it takes is complementing the function above with interaction terms of the type *γ*_*k*_ ⋅ (*b*_*k*_*y*_*i*_). Partial differentiation of *w*(·) w.r.t. then *b*_*k*_ results in *δ*_*k*_ + *γ*_*k*_ ⋅ *y*_*i*_. For example, if *γ*_*k*_ > 0 and *δ*_*k*_ < 0, marginal utility of attribute *k* decreases as a function of income.

###  Marginal rate of substitution and willingness-to-pay

In the course of the experiment, participants need to tradeoff between the different attributes of a scenario. Their preference structure is reflected by the marginal rate of substitution (*MRS*) between two attributes *k* and *m*, given by the ratio of the two respective marginal utilities,

(7) $$\mathrm{MR}S_{k,m}=-\frac{\partial v/\partial b_{k}}{\partial v/\partial b_{m}}\text{.}$$

**Figure 1:**
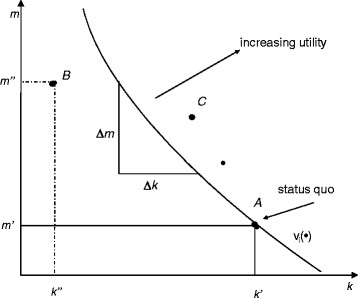
Marginal rate of substitution between two attributes.

This trade-off and its experimental measurement are illustrated by Figure 1.

The point of departure is the status quo, symbolized by point *A*, with much (*k’*) of attribute *k* and little (*m’*) of attribute *m*. Now the respondent is faced with alternative *B*, with only *k”* < *k’* of attribute *k*, but *m”* > *m’* of attribute *m* in return. If the respondent prefers the status quo, it must have higher utility than the alternative. This means that *B* lies below the indifference curve through *A*, causing the respondent to stay with the status quo. However, another alternative given by point *C* would be preferred to the status quo. Clearly, through repeated choices, the indifference curve can be interpolated, with Δ*m*/Δ*k* denoting the *MRS* between the two attributes.

Now redefine attribute *m* as the net income after having paid the price for the good (here, the premium for the health insurance contract). By partially differentiating the indirect utility function with regard to the price attribute, one therefore obtains the (negative of) the marginal utility of income. The *MRS* then indicates how much income an individual is prepared to sacrifice in order to obtain more of attribute *k*. This amounts to the marginal willingness-to-pay for attribute *k,* measured in money (Louviere et al. [12] ch. 3).

## Results

###  The discrete choice experiment in Germany

The choice of relevant attributes describing a health insurance contract is far from clear. However, in Germany the policy debate had been revolving about the following attributes, which also turned out to be ‘important’ in a qualitative pretest.

**Amount of physician choice.** Here, the status quo is free physician choice (see Table 1). One alternative is a physician list established by the health insurer, based on cost and quality criteria. A second alternative is gatekeeping, meaning that a primary care physician must be contacted first in the event of illness. It is only then that the patient can choose a specialist. The third, most restrictive alternative is gatekeeping combined with a list of specialists participating in a network. Again, first the gatekeeping physician must be contacted; in addition however, referrals can only be made to other network physicians (who must take part in quality assurance meetings and continued education).

**Second opinion.** Here, the status quo requires patients to come up with 10 Euro per quarter for every additional physician they contact unless referred by the treating physician. In the alternative, one second opinion per quarter can be had free of charge.

**Additional services or information provided by health insurers.** The status quo is no particular services or information provided. However, when insurers are to offer contracts with new ways of providing care, consumers’ demand for information quite likely increases. Therefore, the alternative scenario provides for a qualified person available on the telephone 24 hours per day for helping to organize medical care and to inform about the seriousness of symptoms.

**Incentives.** Since the insured do not have to fully bear the financial consequences of an illness, they might be tempted to skim on preventive effort or opt for the more costly therapy (Zweifel et al. [13], ch. 6). The status quo is characterized by the absence of any measures designed to counteract these moral hazard effects. A first alternative is a bonus option for no claims. If no health care services (except recommended preventive and screening services) are utilized during a year, there is a premium rebate of 500 Euro. The second alternative is a yearly deductible of 500 Euro, again with the exceptions just mentioned. Third, an insured who proves to have performed preventive activities recommended by the insurer would obtain a bonus such as reimbursement of fees or a free week-end at a spa.

**Increase or decrease of the annual health insurance contribution.** Participants were asked to check their pay schedule in order to calculate their personal share of the total contribution in Euro. The alternatives were increases and decreases of 200, 300, 400, and 500 Euro annually. The higher amounts seem unrealistic; however, they need to be set in a way that respondents sometimes move away from the status quo, generating information about their preferences.

**Table 1:** Status quo quo card (German DCE)

**Your current policy**	
1. Amount of physician choice	Unrestricted
2. Second opinion	10 Euro fee without a referral
3. Additional services provided by insurer	No special services or information provided
4. Incentives	No special incentives
5. Health insurance contribution	Your current annual contribution in Euro ___

The five stated attributes above and their levels can be combined to form scenarios that can be compared to the status quo. There is a total of 512 (=4^2^ · 2^2^ · 8), too many for an experiment. The number of scenarios was reduced using a so-called optimal design using GOSSET (Carlsson and Martinsson [14]). The resulting 24 scenarios were split up in random order into three sets with eight decisions each. Table 2 contains an example of a decision card. The DCE was fielded in September 2005, involving around 1,000 individuals of age 25 and older, all members of statutory health insurance. The experiment was conducted on the basis of a written questionnaire. The sample was drawn from a household panel of 70,000 German households of a marketing agency that also conducted the Dutch survey. The German sample is quite representative of the resident population with respect to age, gender, occupation, and income. However, since respondents repeatedly participate in surveys, there may still be selection effects which are impossible to control for (for more details, see Becker, Brändle and Zweifel [15]). Subscribers to private health insurance were excluded because different product attributes would have been relevant to them.

**Table 2:** Example of a decision card (German DCE)

**Alternative 1**	
1. Amount of physician choice	Physician list
2. Second opinion	10 Euro fee without referral
3. Additional services provided by insurer	Patient counseling provided by insurer
4. Incentives	Bonus for preventive behavior
5. Increase/decrease of health insurance contribution	Reduction by 500 Euro annually
I opt for this alternative	□
I opt for my current policy	□

Choices were analyzed using the Probit model, with the random effects specification as described in equation (5), using STATA (as well as LIMDEP for a consistency check). The only explanatory variables are the (changes in) attributes, making up the so-called core model (see Table 3).

**Table 3:** Estimation results for the core model (attributes only), Germany

**Attribute**	**Expected sign**	**Coefficient**		**Std. error**	**z-value**	**Marg. eff.**
Physician list	-	−0.69588	***	0.06229	−11.17	−0.14019
Gatekeeping	-	−0.23197	***	0.05848	−3.97	−0.05284
Network	-	−0.40916	***	0.05944	−6.88	−0.08896
Second opinion	+	0.16004	***	0.04551	3.52	0.03869
Extra services	+	0.24688	***	0.04378	5.64	0.05968
Bonus no claims	+	0.72262	***	0.06023	12.00	0.19911
Deductible	-	−0.49546	***	0.06607	−7.50	−0.10754
Bonus prev. beh.	+	0.40934	***	0.07948	5.15	0.11204
Contribution	-	−0.00201	***	0.00007	−30.62	−0.00049
Constant	0	−1.00709	***	0.07445	−13.53	

*σ*_*υ*_ = 0.9472 *ρ* = 0.4729.

Log likelihood: −3.074.

χ^2^ (0) = 742.57, Prob > χ^2^ = 0.0000.

*n* = 7,155.

***Coefficient different from zero with error probability < 1 percent.

The typical MC attributes (physician list, gatekeeping, physician network) are hypothesized to be associated with losses of utility on the part of consumers (see the negative entries in the column, ‘Expected sign’). Indeed, all three coefficients are significantly negative. Conversely, a second opinion provided free of charge and additional services provided by the health insurer are valued positively, as predicted. The next two attributes, bonus for no claim and deductible, are of particular interest. One could argue that a bonus for no claims amounting to 500 Euro exposes the insured to the same risk as a fixed deductible of 500 Euro because they will end up paying the first 500 Euro out of pocket in both cases. However, this argument overlooks the fact that a bonus option permits consumers to separate two losses in time that occur simultaneously under the deductible, viz. the health loss and the financial loss caused by the cost of medical care. With a deductible, these two losses are perfectly correlated during a quarter (say). With a bonus option, they are separated in time because consumers can sacrifice their bonus to obtain full coverage, shifting the financial loss to later periods in the guise of a higher premium (Zweifel and Waser [16], ch. 3). Indeed, Table 3 shows that respondents valued the bonus option favorably, while resisting a deductible of the same amount. However, they are also interested in a bonus for preventive behavior. The price attribute has a negative coefficient as predicted and is of very high statistical significance. Finally, the constant is worth commenting. If the core model were completely specified, it should be zero because the (changes in) attributes included account fully for the difference in utility between the status quo and the respective alternative – a rather unlikely event. The negative value of the constant points to status quo bias, reflecting neglected determinants of utility that cause the alternative to be valued less highly *ceteris paribus*. Indeed, 20 percent out of the 1,000 participants never moved away from the status quo, while four percent never made a decision between the status quo and the alternative.

Note also that the estimated marginal effects are intuitive. For example, a physician list lowers the probability of changing in favor of the alternative by an estimated 14 percentage points. Having to first visit a gatekeeping physician of one’s choice is a far less stringent restriction. It is associated with a probability reduction of 5.3 percentage points only. Having to sign up with a physician network comprising also specialists has a lock-in effect, causing the probability of choosing the pertinent alternative to drop by an estimated 8.9 percentage points. Compared to these attributes, a second opinion free of charge and extra services provided by the insurer have less impact (3.9 and 6.0 percentage points, respectively), as one would expect. The one astonishing result is that the bonus for preventive behavior apparently is as important (in absolute value) as a deductible of 500 Euro.

Using equation (7), marginal willingness-to-pay (WTP) values can be calculated from the coefficients displayed in Table 3. The three attributes typical of MC options have all to be compensated (see Table 4). Maximum compensation is required for accepting a physician list, amounting to 346 Euro per year, followed by participation in a physician network (203 Euro), and acceptance of gatekeeping (115 Euro). Obtaining a second opinion free of charge is valued at 80 Euro and extra services provided by the health insurer, at 123 Euro per year. As was to be expected, a bonus for no claims triggers a positive WTP value, whereas a deductible amounting to the same value of 500 Euro would have to be compensated. The difference between the two values is striking, amounting to no less than 605 Euro (=349 – (−246)). Finally, the bonus for preventive effort is valued at 203 Euro annually.

**Table 4:** Marginal willingness-to-pay values for attributes (Germany), Euro/year

**Attribute**	**WTP**	**Significance**	**Std. error**
**Physician list**	−346	***	31.04
**Gatekeeping**	−115	***	29.28
**Network**	−203	***	29.80
**Second opinion**	80	***	22.33
**Extra services**	123	***	22.32
**Bonus no claims**	359	***	30.04
**Deductible**	−246	***	33.51
**Bonus f. prev. beh.**	203	***	37.87
**Contribution**	−500	***	36.49

***WTP different from zero with an error probability of < 1 percent. Standard errors calculated using the delta method.

**Conclusion 1:** In the German DCE, there is clear evidence suggesting that respondents value health insurance attributes in the way one would expect from economic considerations.

In the following, status quo bias will be analyzed in greater detail because of its importance for policy. From Table 4, one can conclude that Germans are unwilling to move away from the status quo unless they are compensated by 500 Euro on average. However, this amount varies with socioeconomic characteristics.

The entries of Table 5 are derived from a comprehensive model that includes interaction terms in the Probit equation as described below equation (6). Using equation (7), one can calculate WTP estimates, with all other characteristics set at their median sample values.

**Table 5:** Group-specific status quo bias (WTP values), Germany

	**WTP values for changing contract**		
	**Value (in Euro)**		**Std. error**
**Women**	−508***		50.05
**Men**	−483***		53.33
Prob > chi2/(chi2)^a)^	0.7392/(0.11)		
**Age < 43**^**b)**^	−329***		50.13
**Age 43 – 59**^**b)**^	−407***		58.98
**Age > 59**^**b)**^	−940***		106.87
**Prob > chi2/(chi2)**^**a)**^	***0.0000/(27.70)***		
**Retired**	−953***		118.96
**Non-retired**	−402***		38.11
Prob > chi2/(chi2)^a)^	***0.0000/(19.45)***		
**East**	−520***		83.85
**West**	−494***		40.46
Prob > chi2/(chi2)^a)^	0.7841/(0.08)		
**City with > 50,000 inh.**	−569***		69.67
**City with < 50,000 inh.**	−473***		42.82
Prob > chi2/(chi2)^a)^	0.2397/(1.38)		
**Education low**^**c)**^	−590***		92.07
**Education medium**^**c)**^	−520***		43.86
**Education high**^**c)**^	−411***		74.72
Prob > chi2/(chi2)^a)^	0.4642/(1.54)		
**Healthy (subjective)**	−297***		54.05
**Ill (subjective)**	−609***		48.98
Prob > chi2/(chi2)^a)^	***0.0000/(18.95)***		
**Non-chronic**	−446***		39.72
**Chronic**	−641***		87.07
Prob > chi2/(chi2)^a)^	***0.0416/(4.15)***		
**No physician visit**^**d)**^	−402***		68.55
**Physician visit**^**d)**^	−533***		42.90
Prob > chi2/(chi2)^a)^	***0.1221/(2.39)***		

^a)^Group-specific values differ with an error probability = Prob > chi2 (chi^2^-value after slash, bold and in italics if difference is significant at 5 percent or better).

^b)^Each of the three age groups contains about 33 percent of observations.

^c)^Individuals with 9, 12, and 18 years of education, respectively.

^d)^At least one physician visit related to illness during the past 12 months.

*** (**, *) WTP values (compensations asked) different from zero with error probability of > 1 (>5, > 10) percent.

While there is no recognizable gender difference, status quo bias does significantly increase with age. Retired persons exhibit maximum status quo bias, amounting to 953 Euro. Residents of Eastern (communist up to 1989) and Western Germany and of cities of different size appear to be homogeneous. Somewhat unexpectedly, education does not seem to have a significant influence, although there is some indication of less status quo bias among the highly educated (who presumably can more easily acquire the information needed to compare an alternative to the status quo). As could be expected however, respondents who subjectively feel in bad health require particularly high compensation for moving away from the status quo, as is true of chronic patients. This is interesting because so-called demand management programs focus on chronically ill persons, who are alleged to value them because of better coordination of therapy. This expectation is not borne out; to the contrary, chronically ill respondents exhibit an especially marked preference for the status quo (for more detail, see MacNeil Vroomen and Zweifel [17]). This is also true of persons who had at least one illness-related physician visit during the past twelve months.

**Conclusion 2:** The German DCE points to marked status quo bias, whose magnitude differs between socioeconomic groups, however.

###  The discrete choice experiment in the Netherlands

A second DCE was performed in the Netherlands in May 2006. By March 2006, every citizen had to have explicitly chosen a health insurance contract. Therefore, respondents had been made to bear the cost of decision associated with the choice of a health insurance policy. While most of the attributes were the same as in the German DCE, three adjustments were necessary. First, pretests suggested a second opinion free of charge to be far less important than expeditious (defined to be within four weeks in the DCE) access to hospital care. In fact, waiting for hospital treatment was a hotly debated issue in the Netherlands at the time. Second, the status quo for physician choice and incentives had to be defined differently. Already before the reform of 2006, physician choice was constrained in that patients had to contact a gatekeeping physician first. Therefore, one of the alternatives became free physician choice. Third, there was already a bonus for no claims under the status quo, attaining a maximum of 255 Euro annually. The survey was conducted by the same marketing agency as in Germany, using a written questionnaire. The 763 respondents of a panel of Dutch households closely matched the resident population [see Becker, Brändle and Zweifel [15] for more details]. Here again, 20 percent never moved away from the status quo; however, only 0.7 percent never made a decision (compared to 4 percent in Germany).

Probit estimates are displayed in Table 6. The three variables relating to physician choice are highly significant and have the predicted sign (recall that the status quo in the Netherlands is gatekeeping). Guaranteed access to hospital care within four weeks is positively valued as predicted, as are additional services provided by the health insurer. However, the transition from the existing bonus option for no claims to an alternative without such an option (or one worth 500 Euro, respectively) is not relevant. What is strongly resisted is a deductible amounting again to 500 Euro annually. A bonus for preventive behavior is valued positively and an increase in the annual contribution, negatively (as expected).

**Table 6:** Estimation results for the core model (contract attributes only), Netherlands

	**Expected sign**	**Coefficient**		**Std. error**	**z-value**	**Marginal effect**
**Free choice of physician**^**a)**^	+	0.22756	***	0.06156	3.70	0.05862
**Physician list**^**a)**^	-	−0.39694	***	0.06703	−5.92	−0.08745
**Network**^**a)**^	-	−0.22051	***	0.06446	−3.42	−0.05086
**Hospital access**	+	0.20267	***	0.05012	4.04	0.04943
**Service insurer**	+	0.16260	***	0.04796	3.39	0.03966
**No bonus option**	-	0.02117		0.05857	0.36	0.00519
**Deductible**	-	−1.18245	***	0.07178	−16.47	−0.22555
**Bonus prev. behav.**	+	0.00038		0.08081	0.00	0.00009
**Contribution**	-	−0.00289	***	0.00010	−27.53	−0.00071
**Constant**	0	−0.74269	***	0.07523	−9.87	

*σ*_*υ*_ = 0,8258 *ρ* = 0,4055.

Log likelihood: −2.541,09.

χ^2^ (0) = 11.179,10, Prob > χ^2^ = 0,0000.

*n* = 5,976.

^a)^Status quo is gatekeeping.

***Coefficient different from zero with error probability < 1 percent.

The estimated marginal effects are reasonable. Free choice of physician is associated with a 5.9 percentage point increase in the probability of choosing the alternative. The transition from gatekeeping to a physician list established by the health insurer serves to decrease this probability by 8.7 percentage points. Thus, a change from free physician choice to such a physician list can be estimated to lower choice probability by 14.7 (=5.9 + 8.7) percentage points. A change to a physician network would have a somewhat smaller effect, amounting to a reduction in probability by 11.0 (=5.1 + 5.9) percentage points. Access to the hospital within four weeks is associated with an increase of 4.9 percentage points in choice probability, followed by additional services provided by insurers (3.9 percentage points). Increasing the bonus option for no claims from 255 to 500 Euro does not seem to affect choice probability. However, the most striking result is that an annual deductible amounting to 500 Euro would cause the likelihood of accepting the alternative to drop by no less than 23 percentage points.

Again, WTP values (shown in Table 7, left-hand side) can be derived from Probit coefficients. Concentrating on the Dutch values, one notes first that changing from gatekeeping to free choice of physician would trigger a WTP value of 79 Euro, while the change to a physician list would require compensation to the tune of 137 Euro per year. Assuming local constancy of the MRS, one can therefore infer that the transition from free physician choice to a physician list would require a compensation of 216 Euro (=79 + 137) annually, compared to 155 Euro (=79 + 76) for the transition to a physician network. These estimates make intuitive sense because a physician list constitutes the harshest restriction, followed by a physician network (with its potential lock-in effect) and followed by gatekeeping (the status quo in the Netherlands). Guaranteed hospital access within four weeks is valued somewhat less, presumably because respondents take the comparatively low likelihood of hospitalization into account. Additional services provided by the health insurer is at the low end with 56 Euro per year, while increasing the bonus option to 500 Euro (or to zero, respectively) and the bonus for preventive behavior have no significant WTP value.

**Table 7:** Marginal willingness-to-pay values for attributes (derived from the core model), Netherlands compared to Germany

	**Δ**^**a)**^	**WTP Netherlands**	**Std. error**	**WTP Germany**	**Std. error**
**Physician list**	Δ	−216***	25.02	−346^b)^	33.04
**Network**		−155***	22.09	−203	29.80
**Hospital access**		70***	17.12	n.a.	n.a.
**Second opinion**		n.a.	n.a.	80	22.33
**Services insurer**	Δ	56***	16.76	234	22.32
**Bonus option**	Δ	−7	20.25	359^c)^	30.04
**Deductible**	Δ	−409***	27.37	−246	33.51
**Bonus prev. beh.**	Δ	0	27.95	203	37.87
**Constant**	Δ	−256***	25.87	−500	36.49

Values in Euro per year.

^a)^Difference between Germany and the Netherlands significant at the 5 percent level or better.

^b)^Transferred from Table 6. For comparison purposes, the figures for the Netherlands take free choice of physician as the benchmark.

^c)^WTP for increasing the bonus for no claims from 255 to 500 Euro in the Netherlands.

***WTP different from zero with an error probability of < 1 percent.

By way of contrast, a 500 Euro deductible would have to be compensated by no less than 409 Euro to be accepted. This points to an extreme degree of risk aversion, since a full 42 percent of Dutch respondents stated that they had no illness-related physician visit during the previous year. Conservatively assuming that healthcare expenditure can be either 0 or 500 Euro, one obtains an expected value of 290 Euro (=0.42 · 0 + 0.58 · 500), with variance *Var*(*W*) = 60,900. Applying the Arrow-Pratt formula (Arrow [18]),

(8) $$\mathrm{WT}P_{s}=\frac{1}{2}\mathrm{RA}\cdot \mathrm{Var}\left(W\right)\text{,}$$

with *WTP*_*s*_ denoting *WTP* for safety. Since it amounts to 119 Euro (=409 – 290), one obtains a coefficient of absolute risk aversion RA = 0.0039. With average wealth among respondents set at *W* = 10,000 Euro (the concentration of financial wealth is high among the Dutch, with the top 1 percent having a 25 percent share, see Gowling [19]), the coefficient of relative risk aversion RR amounts to^a^

(9) RR=W⋅RA≅39.

This is an extremely high value in international comparison (see e.g. Barsky et al. [20] for U.S. values, which do not exceed 6). It can also be interpreted as the elasticity of *WTP*_*s*_ w.r.t. wealth, where aggregate values around 1.5 are common in industrial countries (Szpiro [21]).

Again, group-specific WTP values in the Dutch sample are derived (see Table 8, left-hand side). There is no evidence of a gender-specific difference, while status quo bias clearly increases with age and retired status. Prior to the 2006 reform, a distinction existed between privately and legally insured (in a way similar to Germany, where high-wage earners can opt out of the statutory scheme). With the reform, this distinction was lifted. However, there is no significant difference between the two groups of insured. Community size does not matter either; however, there is evidence suggesting that highly educated respondents have a more marked status quo bias than others, which is true also of those who feel ill subjectively, the chronically ill, and those with an illness-related physician visit during the past twelve months (for more details, see MacNeil Vroomen and Zweifel [17]).

**Table 8:** Group-specific estimates of status quo bias, Netherlands compared to Germany

	**WTP values for changing contract**			
	**Netherlands**	**Std. error**	**Germany**^**e)**^	**Std. error**
**Women**	−226***	34.65	−508***	50.05
**Men**	−292***	38.90	−483***	53.33
**Prob > chi2/(chi2)**^**a)**^	0.2020/(1.63)		0.7392/(0.11)	
**Age < 41**^**b)**,**e)**^	−162***	35.56	−329***	50.13
**Age 41 – 55**	−234***	42.63	−407***	58.98
**Age > 55**	−479***	70.97	−940***	106.87
**Prob > chi2/(chi2)**^**a)**^	***0.0559/(5.81)***		***0.0090/(27.70)***	
**Retired**	−456***	85.83	−953***	118.96
**Non-retired**	−221***	26.93	−402***	38.11
**Prob > chi2/(chi2)**^**a)**^	***0.0089/(6.84)***		***0.0000/(19.45)***	
**Privately insured**^**e)**^	−293***	46.15	n.a.	n.a.
**Legally insured**	−240***	31.23	n.a.	n.a.
**Prob > chi2/(chi2)**^**a)**^	0.3403/(0.91)		0.7841/(0.08)	
**City with > 50,000 inh.**^**a)**^	−241***	34.87	−569***	69.67
**City with < 50,000 inh.**^**a)**^	−281***	38.95	−473***	42.82
**Prob > chi2/(chi2)**^**a)**^	0.4511/(0.57)		0.2397/(1.38)	
**Education low**^**c)**^	−212***	37.60	−590***	92.07
**Education medium**^**c)**^	−261***	50.87	−520***	43.86
**Education high**^**c)**^	−336***	51.46	−411***	74.72
**Prob > chi2/(chi2)**^**a)**^	0.1521/(3.77)		0,4642/(1.54)	
**Healthy (subjective)**	−164***	33.99	−297***	54.05
**Ill (subjective)**	−325***	38.33	−609***	48.98
**Prob > chi2/(chi2)**^**a)**^	***0.0017/(9.88)***		***0.0000/(18.95)***	
**Non-chronic**	−225***	28.67	−446***	39.72
**Chronic**	−351***	58.44	−641***	87.07
**Prob > chi2/(chi2)**^**a)**^	***0.0542/(3.71)***		***0.0416/(4.15)***	
**No physician visit**	−204**	37.36	−402***	68.55
**Physician visit**^**d)**^	−297***	36.05	−533***	42.90
**Prob > chi2/(chi2)**^**a)**^	***0.0722/(3.23)***		***0.1221/(2.39)***	

^a)^Group-specific values differ with an error probability = Prob > chi2 (chi^2^-value after slash, bold and in italics if difference is significant at 5 percent or better).

^b)^Age groups are < 43, 43–59, and > 59 in the German sample to contain about 55 percent of observations.

^c)^Individuals with 9, 12, and 18 years of education, respectively.

^d)^At least one physician visit related to illness during the past 12 months.

*** (**, *) WTP values (compensations asked) with error probability of > 1 (>5, > 10) percent different from zero.

**Conclusion 2:** The Dutch DCE suggests that a change in the bonus option for no claims and a bonus for preventive behavior are irrelevant to consumers. The other estimated WTP values are significant and in accordance with economic considerations.

## Discussion

The evidence presented permits to address the two research questions posed in the Background section. They both involve a comparison between the two countries, which can be performed using Tables 7 and 8. Table 7 shows the relative importance of product attributes in the Netherlands compared to Germany. With regard to the attributes, recall that WTP values for the Netherlands are measured as deviations from a counterfactual status quo ‘free choice of physician’. The following statements are based on a joint dataset containing only overlapping attributes. A dummy variable taking on the value 1 if the observation relates to the Netherlands is interacted with the explanatory variables of the core model. A ‘Δ’ indicates that this dummy variable is statistically significant.

Thus, Dutch respondents would have to be compensated less for accepting a physician list created by health insurers than their German counterparts. It seems that they are already used to insurers having influence on physician choice. However, the transition to a physician network would have to be compensated to the same degree in the two countries. On the other hand, in the Dutch sample extra services provided by the health insurer are valued less than in the German sample.

The most salient differences concern the assessment of a bonus for no claims and of a deductible. The Dutch sample exhibits an insignificant willingness to pay for a change away from the status quo value of 255 Euro, whereas in the German sample there is a substantial WTP for increasing it to 500 Euro. With regard to the 500 Euro deductible, the Dutch appear to be far more risk averse than the Germans in that they would have to be compensated by no less than 409 Euro, compared to 246 Euro in the German sample [see equation (9) again]). One explanation of this difference may that the Dutch are exposed to an additional risk that does not concern the Germans. This is the income risk associated with a sickness episode, which is usually neglected by health economists, as emphasized in Zweifel and Manning [22]. Indeed, in response to the ´Dutch disease´ of the early 1990s, the Dutch government implemented reforms designed to reduce the incidence of sick leave. The new Sickness Benefits Act of 1998 obliged employers to hire a private certified agency to develop a rehabilitation program within 90 days of sick leave. This was complemented by the Improved Gatekeeper’s Act in 2002, which required employers to evidence ‘sufficient’ effort during the first 52 weeks of a sick leave spell before being able to apply for long-term benefits (paid to them rather than their workers) (Bockting [23]). By way of contrast, there is no monitoring of sick leave pay in Germany; in addition, the full wage continues to be paid by employers for the first six weeks, after which time the sick funds take over. Up to 2006, this generous scheme applied to blue-collar workers only; from then on, it was extended to cover white collars as well. For the Dutch, an increased bonus for no claims and especially a deductible therefore amounts to an accumulation of two risks, which they seek to avoid. In the terms of Eeckhoudt and Schlesinger [24], the Dutch would face a higher ´pain of risk bearing´ than the Germans.

Finally, a bonus for preventive behavior does not trigger willingness to pay at all in the Dutch sample but is valued with a remarkable 203 Euro per year in Germany. Since the WTP values differ between the two samples with those for the physician network as the only exception, one may draw

**Conclusion 3:** Question Q1 can be answered as follows. Whereas most of comparable attributes of a health insurance contract are valued the same qualitatively, almost all WTP values differ in magnitude, pointing to differences in preference between the two countries.

It should be borne in mind, however, that the differences found can also be caused by differences in the status quo or in excluded non-overlapping attributes [the utility function may not be additively separable as assumed in equation (6)].

The last row of Table 7 contains a preliminary answer to the second question. In the Dutch sample, status quo bias amounts to just about one-half of the German value. However, the determinants of status quo have very much the same effects in the two countries (see Table 8). Higher age, retirement status, being subjectively ill, being chronically ill, and having an illness-related physician visit during the previous twelve months all cause it to be higher than among the remainder of the sample (no significance tests are available for checking differences in gradients). On the other hand, neither gender nor community size matter in the two countries. There is one notable difference in that higher education in the Netherlands goes along with higher status quo bias, whereas the tendency is almost the reverse in Germany. In all, the evidence supports

**Conclusion 4:** The answer to question Q2 is that the requirement to explicitly choose a health insurance policy imposed by the Dutch government in the context of the 2006 reform may well have served to reduce status quo bias with regard to future reforms, without however eliminating group-specific differences.

As a final piece of evidence, the standard errors shown in Table 8 can be compared. They are consistently lower in the Dutch than in the German sample, although the Dutch DCE involved only 760 rather than 1,000 respondents. Given identical sample size, they should even be 13 percent lower [(760/1,000)^½^ = 0.87]. Apparently, the 2006 reform had caused citizens to bear the information cost associated with deciding between health insurance contracts by the time the DCE was fielded (May 2006). Of course, there is still the alternate explanation that the Dutch have more homogenous preferences with regard to health insurance than the Germans.

## Conclusions

This contribution is one of the few that seek to compare preferences with regard to health insurance across national borders. The objective was to find out whether the Dutch might value attributes of health insurance and provision of health care differently than the Germans. The comparison is based on two Discrete Choice Experiments (DCEs) performed in Germany (with no effective reform) and in the Netherlands right after the 2006 reform, which made citizens explicitly choose their health insurance. One finding is that important contract attributes are valued in the same qualitative way by the two populations. Specifically, Managed Care-type features such as a physician list established by the health insurer, gatekeeping, and adherence to a physician network must be compensated in both countries. Conversely, additional services provided by the health insurer trigger positive WTP whereas an annual deductible of 500 Euro would have to be highly compensated to be accepted. However, bonuses for proven preventive effort and no claims are received favorably only in Germany, not in the Netherlands. Differences also arise with regard to the magnitudes of WTP values. Notably, a 500 Euro deductible has to be compensated almost twice as much in the Netherlands than in Germany, likely because of an income risk due to illness confronting the Dutch. Therefore, one first has to conclude that there is evidence of differences in the preference structure of the two populations. Second, however, there is a striking difference in terms of status quo bias, which requires only one-half as much compensation in the Netherlands compared to Germany. Therefore, the requirement to specifically choose a health insurance policy imposed by the Dutch government in the context of the 2006 reform may well have been effective in reducing status quo bias, potentially facilitating future reforms of health insurance.

## Endnote

^a^There is an alternative way of deriving the RA estimate, by using an indifference relation involving a Euro 500 deductible and the required compensation in the same way as the Arrow-Pratt formula is derived (Fred Schroyen, private correspondence). However, while involving an additional Taylor approximation, it leads to a very similar value (*RA* = 0.0049).

## Competing interests

The authors declare that they have no competing interests.

## Authors’ contributions

KLB carried out the preparation of the surveys and discrete-choice-experiments and did the statistical analysis (descriptive statistics and econometric estimation) of the data. KLB and PZ jointly discussed the results and drew the conclusions. KLB prepared a first draft and publication of final report on the entire analysis, written in German which was reviewed by PZ. PZ prepared the paper (English version) for this journal. All authors read and approved the final manuscript.
